# Molecular Weight Distribution Control for Polymerization Processes Based on the Moment-Generating Function

**DOI:** 10.3390/e24040499

**Published:** 2022-04-01

**Authors:** Jianhua Zhang, Jinzhu Pu, Mifeng Ren

**Affiliations:** 1 State Key Laboratory of Alternate Electrical Power System with Renewable Energy Sources, North China Electric Power University, Beijing 102206, China; zjh@ncepu.edu.cn; 2 School of Control and Computer Engineering, North China Electric Power University, Beijing 102206, China; 1162127005@ncepu.edu.cn; 3 College of Electrical and Power Engineering, Taiyuan University of Technology, Taiyuan 030024, China

**Keywords:** molecular weight distribution, moment-generating function, B-spline

## Abstract

The molecular weight distribution is an important factor that affects the properties of polymers. A control algorithm based on the moment-generating function was proposed to regulate the molecular weight distribution for polymerization processes in this work. The B-spline model was used to approximate the molecular weight distribution, and the weight state space equation of the system was identified by the subspace state space system identification method based on the paired date of B-spline weights and control inputs. Then, a new performance criterion mainly consisting of the moment-generating function was constructed to obtain the optimal control input. The effectiveness of the proposed control method was tested in a styrene polymerization process. The molecular weight distribution of the styrene polymers can be approximated by the B-spline model effectively, and it can also be regulated towards the desired one under the proposed control method.

## 1. Introduction

Polymers are high molecular compounds consisting of macromolecules with different chain lengths [1]. The properties of polymers are affected by their molecular weight distribution (MWD) [2,3,4,5,6], which can be measured directly or modelled mathematically [7,8,9]. The production of polymers is widespread and long-standing in industrial processes; different applications need different specifications for the polymers [10]. To meet these demands, many performance indices consisting of polydispersity and average molecular weight have been proposed. Under these indices, the control objective is commonly dealt with as an optimization problem, which has been investigated in many studies [11,12,13,14,15,16,17,18,19,20,21]. Though satisfactory control performance could be obtained in these studies, these indices cannot fully characterize the entire molecular weight distribution of polymers.

However, the shape of the molecular weight distribution of polymers is essential in many polymerization processes, such as paints and paper coatings [22,23], and the average molecular weight and polydispersity index is unable to reflect the characteristics of the MWD when the distribution is non-Gaussian. To solve this problem, the stochastic distribution control (SDC) method can be proposed for molecular weight distribution shaping in polymerization processes. The SDC method has been applied to many industrial processes, such as distribution control of the flame temperature in furnace systems [24,25], particulate process control in powder industries [26,27], distribution control of bubble size in flotation processes [28], distribution control of crystal size in crystallization processes [29,30,31] and power probability density function control in nuclear research reactors [32].

Unlike the methods based on performance indices consisting of polydispersity and average molecular weight, the entire molecular weight distribution (MWD) of the polymerization process is able to be regulated towards the target MWD under the SDC method.

Since the MWD cannot be easily used in the MWD shape control of polymerization processes directly, the dynamic modelling of the output MWD as the control input is necessary. B-spline models can be utilized to approximate the output MWD [33]. The main advantage of the B-spline MWD model is the decoupling of the time domain, the MWD definition domain in the formulation and the obtained weights model of the MWD, which can be made mathematically equivalent to any existing physical models for MWD systems subjected to a pre-specified, small modelling error. Although several B-spline-based MWD models have been developed [34,35,36,37], the linear B-spline MWD model is applied in this paper due to its computational simplicity, which can reduce the computational time [38].

Next, a dynamic weights model can be established to make the subsequent control problem easier to manage. Inspired by Greś and colleagues [39], the subspace identification method is used to obtain the state space model between the weight vector of the B-spline model and the control input. Based on the input and weight vector data of the B-spline model, the state space model parameters of the system can be obtained through the row subspace and column subspace mapped by the Hankel matrix. Canonical variate analysis, multivariable output error state space and numerical subspace state space system identification are three influential subspace identification methods. Compared to the traditional identification method, numerical subspace state space system identification (N4SID) requires less calculation and satisfies numeric stability [40]; therefore, the N4SID was adopted in this paper.

Based on the weights model, control algorithms can be derived by minimizing performance criteria, which indicate the difference between the output MWD and the target MWD. As the characteristic of the MWD can be reflected by its moment-generating function (MGF), a new performance criterion using MGF based on the state space model was proposed in this paper. Compared to the traditional performance criteria [33,34,35,36,37,38], the new criterion needs no integral operation on the quadratic error and has less dependence on the regulation of the criterion weights. The effectiveness of the proposed control method was introduced in a styrene polymerization process. 

In this paper, a novel control algorithm based on the moment-generating function for polymerization processes was presented. The B-spline model was used to approximate the MWD, and N4SID was introduced for system modelling. Then, a performance criterion mainly consisting of the moment-generating function of the MWD was constructed, and the control law could be obtained by minimizing the performance criterion. This control algorithm was applied to a styrene polymerization process and simulation experiment to verify its effectiveness.

## 2. B-Spline Model and Subspace Identification Method

**Figure 1:**
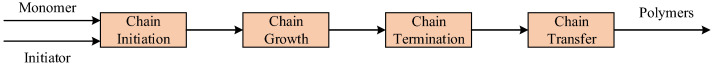
Chain reaction polymerization process.

For a polymerization process, the monomer is transformed into a polymer through a series of chemical reactions. The free radical polymerization process includes chain initiation, chain growth, chain transfer and chain termination. The reaction forming monomer free radicals is called the chain initiation reaction, and it is the key to controlling the rate throughout the polymerization reaction. An initiator added to the monomer is heated to generate initiating radicals, and once the free radical monomer is generated, it will immediately be added to the second monomer to generate a free radical chain containing two monomer units. The activity of the free radical chain does not decay; it will immediately be added to the third monomer and subsequent monomers in the chain. Then, the degree of polymerization of chain radicals increases rapidly; these reactions that increase the degree of polymerization are called chain growth reactions. The binding reaction of free radicals is called a termination reaction, which has two forms: coupling and disproportionation. In addition, the chain free radicals undergo a transfer reaction with monomers, initiators or formed macromolecules, whereby a polymer chain is terminated, and a new radical capable of propagating is formed. The schematization of proposed reaction mechanisms is shown as Figure 1.

**Figure 2:**
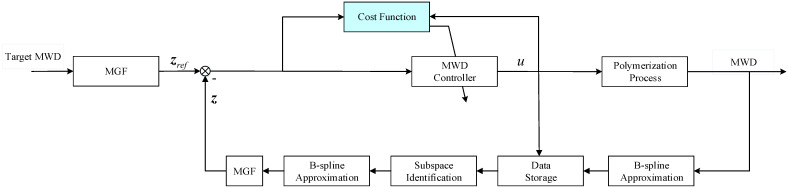
Diagram of the MWD control system for the polymerization process.

The dynamic MWD of the polymerization process can be obtained by the MWD model described in [33] or measured directly through online techniques, and it cannot be easily used in MWD shape control directly. Therefore, a B-spline approximation model is established through the obtained MWD data. Through the weight data of the B-spline model and the control input, the output MWD model can be identified by the N4SID method, which is used in MWD control described later. The schematic diagram of the proposed control algorithm is shown in Figure 2. By using the moment-generating function (MGF), the pseudo-state vector of the target MWD and approximated MWD can be obtained as $z_{ref}$ and z, respectively. The control input, u, is regulated by the MWD controller for the shaping of output MWD. 

### 2.1. B-Spline Approximation for Output MWD

Consider a polymerization process where $u_{k}\in {ℜ}^{d\times 1}$ is the manipulated variable to control the shape of MWD, defined as γ(y,u(k)). This can be approximated by the B-spline neural network

(1) $$\gamma (y,u_{k})=\sum_{i=1}^{n}\omega_{i}(u_{k})B_{i}(y)+e_{0}$$

where $u_{k}$ represents the control input at time instant k, $B_{i}(y)(i=1,\cdots ,n)$ stands for the basis functions which have been pre-designed on the domain of [a,b], *n* represents the number of basis functions and $\omega_{i}(u_{k})$ denotes the expansion weight. $e_{0}$ is the approximation error which can be ignored for expression simplification. 

Since the integration of $\gamma (y,u_{k})$ is equal to 1, the number of independent expansion weights is *n* − 1, and the remaining expansion weight can be expressed by other expansion weights. 

Defining

$$b_{i}={\int_{a}^{b}B_{i}(y)dy}_{\;}(i=1,2,\cdots ,n-1)$$

$$L(y)=\frac{B_{n}(y)}{b_{n}}$$

$$C_{i}(y)=B_{i}(y)-\frac{b_{i}}{b_{n}}B_{n}{\left(y\right)}_{\;}(i=1,2,\cdots ,n-1)$$

$$C(y)=[C_{1}(y),C_{2}(y),\cdots C_{n-1}(y)]$$

$$v_{k}={\left[w_{1}(u_{k}),w_{2}(u_{k}),\cdots w_{n-1}(u_{k})\right]}^{T}$$

Then, the MWD $\gamma (y,u_{k})$ can be reformed as

(2) $$\gamma (y,u_{k})=C(y)v_{k}+L(y)$$

Multiplying $C{\left(y\right)}^{T}$ to both sides of Equation (2) and carrying out an integral operation over the definition domain, the expansion weights vector $v_{k}$ can then be calculated as

(3) $$v_{k}={\left[\int_{a}^{b}C{\left(y\right)}^{T}C(y)dy\right]}^{-1}\int_{a}^{b}C{\left(y\right)}^{T}[\gamma (y,u_{k})-L(y)]dy$$

Since the dynamics of the output MWD can be represented by Equation (1), we assume that the input $u_{k}$ and expansion weights vector $v_{k}$ satisfy the following state space form:

(4) $$\left\{ \begin{array}{l} x_{k}=Ax_{k-1}+Bu_{k-1}\\ v_{k}=Cx_{k}+Du_{k} \end{array}\right.$$

where $x_{k}\in {ℜ}^{\left(n-1\right)\times 1}$ denotes the state vector and A, B, C and D stand for the coefficient matrices of the system, which can be identified by the subspace state space system identification method in the next part.

### 2.2. Subspace State Space System Identification

The relationship between control input $u_{k}$ and expansion weight vector $v_{k}$ can be represented by Equation (4). This paper employs the N4SID method to identify the coefficient matrices A, B, C and D [40]. Compared with traditional methods, N4SID requires less calculation and has a high modelling accuracy.

To save computing space and speed up computer processing, some Hankel matrices are constructed as follows:

(5) $$U_{0|i-1}=\left(\begin{array}{cccc} u_{0} & u_{1} & \cdots & u_{j-1}\\ u_{1} & u_{2} & \cdots & u_{j}\\ \vdots & \vdots & \ddots & \vdots \\ u_{i-1} & u_{i} & \cdots & u_{2i+j-2} \end{array}\right)$$

(6) $$V_{0|i-1}=\left(\begin{array}{cccc} v_{0} & v_{1} & \cdots & v_{j-1}\\ v_{1} & v_{2} & \cdots & v_{j}\\ \vdots & \vdots & \ddots & \vdots \\ v_{i-1} & v_{i} & \cdots & v_{2i+j-2} \end{array}\right)$$

where i is the row number that should be larger than the order of the identified system, j is the column number, and 2i+j−2 should be smaller than the length of the data. 

To simplify the derivation of the N4SID method, some matrices of input and expansion weights are defined as $U_{p}=U_{0|i-1}$, $U_{f}=U_{i|2i-1}$, $V_{p}=V_{0|i-1}$ and $V_{f}=V_{i|2i-1}$.

Similarly, the state matrices can be defined as

(7) $$X_{p}=[x_{1}\;x_{2}\cdots x_{j}]$$

(8) $$X_{f}=[x_{i+1}\;x_{i+2}\cdots x_{i+j}]$$

The generalized observability matrix ($\Gamma_{i}$) and generalized controllability matrix ($\Delta_{i}$) of the identified system are shown as

(9) $$\Gamma_{i}={\left[C\;CA\;CA^{2}\cdots CA^{i}\right]}^{T}$$

(10) $$\Delta_{i}=[A^{i-1}B\;A^{i-2}B\cdots B]$$

A Toeplitz matrix is defined as

(11) $$H_{i}=\left(\begin{array}{ccccc} D & 0 & 0 & \cdots & 0\\ CB & D & 0 & \cdots & 0\\ CAB & CB & D & \cdots & 0\\ \cdots & \cdots & \cdots & \cdots & \cdots \\ CA^{i-2}B & CA^{i-3}B & CA^{i-4}B & \cdots & D \end{array}\right)$$

Then, the state space Equation (4) can be reformulated by the Hankel matrices as

(12) $$V_{p}=\Gamma_{i}X_{p}+H_{i}^{}U_{p}$$

(13) $$V_{f}=\Gamma_{i}X_{f}+H_{i}^{}U_{f}$$

(14) $$X_{f}=A^{i}X_{p}+\Delta_{i}U_{p}$$

Substituting Equations (12) and (13) to Equation (14), $X_{f}$ can be represented as

(15) $$\begin{array}{cc} X_{f} & =A^{i}X_{p}+\Delta_{i}U_{p}=A^{i}\left(-\Gamma_{i}^{†}H_{i}^{}U_{p}+\Gamma_{i}^{†}V_{p}\right)+\Delta_{i}U_{p}\\ & =L_{p}W_{p} \end{array}$$

where $W_{p}={\left[\begin{array}{cc} U_{p} & V_{p} \end{array}\right]}^{T}$, $L_{p}=\left[\begin{array}{cc} \Delta_{i}-A^{i}\Gamma_{i}^{†}H_{i}^{} & A^{i}\Gamma_{i}^{†} \end{array}\right]$ and $\Gamma_{i}^{†}$ is the Moore–Penrose pseudo-inverse of the generalized observability matrix.

Substituting Equation (15) to (13), $V_{f}$ can be denoted as

(16) $$V_{f}=\Gamma_{i}L_{p}W_{p}+H_{i}U_{f}$$

Making an oblique projection of $V_{f}$ to $W_{p}$ along the direction of $U_{f}$

(17) $$\begin{array}{cc} O_{i} & =V_{f}{/}_{U_{f}}W_{p}=\Gamma_{i}L_{p}W_{p}\\ & =\Gamma_{i}X_{f} \end{array}$$

where $O_{i}$ is the oblique projection.

Based on the assumption that System (4) is observable and controllable, $rank\left(O_{i}\right)=n$, and then the singular value decomposition of $O_{i}$ can be shown as

(18) $$O_{i}=USV^{T}=\left[\begin{array}{cc} U_{1} & U_{2} \end{array}\right]\left[\begin{array}{cc} S_{1} & 0\\ 0 & S_{2} \end{array}\right]\left[\begin{array}{c} V_{1}^{T}\\ V_{2}^{T} \end{array}\right]$$

where $S_{1}$ denotes a diagonal matrix consisting of singular values far greater than 0 and $S_{2}$ denotes a diagonal matrix composed of singular values close to 0.

From Equations (17) and (18), $\Gamma_{i}$ and $X_{f}$ can be estimated as

(19) $$\Gamma_{i}=U_{1}S_{1}^{1/2}$$

(20) $${\hat{X}}_{f}=S_{1}^{1/2}V_{1}^{T}$$

Then, the coefficient matrices of System (4) can be obtained by least squares (LS)

(21) $$[A\;B\;C\;D]=\arg_{}\min_{A\;B\;C\;D}{\left|\left(\begin{array}{c} {\hat{X}}_{f,k+1}\\ V_{i} \end{array}\right)-\left(\begin{array}{cc} A & B\\ C & D \end{array}\right)\left(\begin{array}{c} {\hat{X}}_{f,k}\\ U_{i|i} \end{array}\right)\right|}_{F}^{2}$$

Therefore, the estimated expansion weights vector ($v_{k}$) can be obtained by

(22) $$\left\{ \begin{array}{l} {\hat{x}}_{k}=Ax_{k-1}+Bu_{k-1}\\ {\hat{v}}_{k}=C{\hat{x}}_{k}+Du_{k} \end{array}\right.$$

where ${\hat{v}}_{k}$ is the estimated expansion weights vector, and ${\hat{x}}_{k}$ is the estimated state variable vector of the dynamic weights model.

## 3. MWD Shaping Control Algorithm Using MGF for Polymerization Processes

We can note that the MWD is uniquely determined by its moment-generating function. We can carry out a sampling operation on the moment-generating function of the MWD, and the complete information of the MWD can be reflected if the sampling points are sufficiently large. The shape of the MWD approaches the target MWD when the sampling points of the output MWD approach those of the target MWD. Then, a performance criterion using the moment-generating function can be constructed based on the identified dynamic weights model, and the control algorithm can be derived by minimizing the criterion.

### 3.1. Moment-Generating Function

For the traditional performance criterion [33,34,35,36,37,38], if the integral value of the quadratic error between the output MWD and target MWD is very small, then the regulation of criterion weights is critical. In order to reduce the impact of criterion weights on control performance, a moment-generating function is proposed in this section to construct the performance criterion of the proposed control method. The moment-generating function can be represented as

(23) $$M_{k}\left(\xi \right)=\int_{-\infty }^{\infty }\exp\left(\xi y\right)\gamma (y,u_{k})dy$$

where $\xi \in R^{1}$ and $\gamma (y,u_{k})$ is the output MWD at time *k*.

By expanding (23), the following equation can be obtained as follows:

(24) $$M_{k}\left(\xi \right)=1+\xi m_{1}+\frac{\xi^{2}m_{2}}{2!}+\ldots +\frac{\xi^{n}m_{s}}{s!}+\ldots$$

where $m_{s}$ denotes the *s*th moment.

For the convenience of the following calculation, the cumulant-generating function $\phi_{k}\left(\xi \right)$ can be defined as

(25) $$\phi_{k}\left(\xi \right)=logM_{k}\left(\xi \right)$$

The estimated value of $\phi_{k}\left(\xi \right)$, denoted as ${\hat{\phi }}_{k}\left(\xi \right)$, can be calculated utilizing the obtained output MWD. By selecting $\xi_{1},\xi_{2},\ldots ,\xi_{m}$, a pseudo-state vector ($z_{k}$) can be denoted as follows:

(26) $$z_{k}(\xi )={\left[{\hat{\phi }}_{k}\left(\xi_{1}\right),{\hat{\phi }}_{k}\left(\xi_{2}\right),\ldots ,{\hat{\phi }}_{k}\left(\xi_{m}\right)\right]}^{T}$$

Note that enough characteristics of the output MWD can be reflected if *m* is sufficiently large.

Similarly, the pseudo-state vector of the target MWD can be denoted as

(27) $$z_{ref}(\xi )={\left[{\hat{\phi }}_{k}\left(\xi_{1}\right),{\hat{\phi }}_{k}\left(\xi_{2}\right),\ldots ,{\hat{\phi }}_{k}\left(\xi_{m}\right)\right]}^{T}$$

Then, the following performance criterion can be constructed for the controller design:

(28) $$J_{k}={\left(z_{k}-z_{ref}\right)}^{T}Q_{}\left(z_{k}-z_{ref}\right)+\frac{1}{2}u_{k}{}^{T}R_{}u_{k}$$

where Q and R denote the weights, and the second term denotes energy constraints on the control input.

The first term on the right side of Equation (28) can be reformed as

(29) $${\bar{J}}_{k}={\left(z_{k}-z_{ref}\right)}^{T}Q_{}\left(z_{k}-z_{ref}\right)$$

which indicates the difference between the output MWD and target MWD.

### 3.2. Control Algorithm

As shown in Performance Criterion (28), it is obvious that the output MWD approaches the target MWD when the performance criterion decreases. In practical polymerization processes, the constraint on the control input is usually taken into consideration in the case of actuator saturation. 

Then, the optimal control input can be solved as follows

(30) $$\{\begin{array}{cc} {u_{k}}^{*}=arg\;\underset{u_{k}}{min}J_{k}= & arg\;\underset{u_{k}}{min}\left({\left(z_{k}-z_{ref}\right)}^{T}Q_{}\left(z_{k}-z_{ref}\right)+\frac{1}{2}u_{k}{}^{T}R_{}u_{k}\right)\\ & s.t.\;U_{\min}<u_{k}<U_{\max} \end{array}$$

where $U_{\min}$ and $U_{\max}$ stand for the lower and upper bounds of the control input, respectively.

Since the analytical solution of the nonlinear programming optimization problem, (30), cannot be easily solved, the control input can be obtained by quadratic programming in this paper.

## 4. Simulation Study

In this section, the performance of the proposed controller was tested in a styrene polymerization process. Styrene was the monomer for polymerization, and azobisisobutyronitrile was used as the initiator. The monomer and the initiator were blended and pumped into a tank reactor where the polymerization occurred, after which the styrene polymers were produced. Since the ratio of the monomer to the initiator is essential and critical to the polymerization process in the reaction tank, the ratio of the flow rate of the monomer to the sum flow rate, defined as c, is regarded as the manipulated variable in the MWD control system.

### 4.1. Modelling of the Molecular Weight Distribution

Since the molecular weight distribution in the styrene polymerization process is not easily measured directly, dynamic modelling of the molecular weight distribution in the styrene polymerization process is presented in this section.

Under the assumption that the sum flow rate into the tank reactor is invariant, the dynamic model of molecular weight distribution can be established by following the work in [33]. To uphold conciseness of the description, only the major differential equations are presented.

The concentrations in the tank reactor can be described by the following mass balance equations:

(31) $$\frac{dC_{I}}{dt}=\left(C_{I0}-C_{I}\right)/\kappa -K_{d}C_{I}$$

(32) $$\frac{dC_{M}}{dt}=\left(C_{M0}-C_{M}\right)/\kappa -2K_{i}C_{I}-\left(K_{p}+K_{ct}\right)M\phi_{0}$$

where $C_{I}$ and $C_{M}$ stand for the concentrations of azobisisobutyronitrile and styrene, respectively. $C_{I0}$ and $C_{M0}$ denote the initial concentrations of azobisisobutyronitrile and styrene, respectively. κ is a constant which represents the average residential time in the tank reactor. $K_{d}$, $K_{i}$, $K_{p}$ and $K_{ct}$ stand for different rate constants in the polymerization process. $\phi_{0}$ denotes the radical concentration.

Through the use of the generation function technique, the following differential equations can be deduced:

(33) $$\frac{d\phi_{0}}{dt}=\phi_{0}/\kappa +2K_{i}C_{I}-K_{t}\phi_{0}{}^{2}$$

(34) $$\frac{d\phi_{1}}{dt}=-\phi_{1}/\kappa +2K_{i}C_{I}+K_{p}\phi_{0}M-K_{t}\phi_{0}\phi_{1}+K_{ct}M\left(\phi_{0}-\phi_{1}\right)$$

(35) $$\frac{d\phi_{2}}{dt}=-\phi_{2}/\kappa +2K_{i}C_{I}+K_{p}M\left(\phi_{0}+2\phi_{1}\right)-K_{t}\phi_{0}\phi_{2}+K_{ct}M\left(\phi_{0}-\phi_{2}\right)$$

where $\phi_{1}$ and $\phi_{2}$ are leading moments for radicals, and $K_{t}$ stands for the termination rate constant.

Similarly, the differential equations concerning the leading moments for dead polymers can also be derived as

(36) $$\frac{dG_{0}}{dt}=G_{0}/\kappa +K_{ct}C_{M0}\phi_{0}+0.5K_{t}\phi_{0}{}^{2}$$

(37) $$\frac{dG_{1}}{dt}=-G_{1}/\kappa +K_{ct}C_{M0}\phi_{1}+K_{t}\phi_{0}\phi_{1}$$

(38) $$\frac{dG_{2}}{dt}=-G_{2}/\kappa +K_{ct}C_{M0}\phi_{2}+K_{t}\phi_{0}\phi_{2}+K_{t}\phi_{1}{}^{2}$$

where $G_{0}$, $G_{1}$ and $G_{2}$ stand for the leading moments for dead polymers.

Then, the MWD of the polymers can be described by a Schultz–Zimme distribution function, shown as

(39) $$f_{sz}(y)=\frac{{\bar{b}}^{\bar{b}}y^{\bar{b}-1}\exp\left(-\bar{b}y/F_{z}\right)}{F_{z}{}^{\bar{b}}\Lambda \left(y\right)}$$

where y denotes the chain length and

$$\bar{b}=\frac{G_{1}{}^{2}}{G_{0}G_{2}-G_{1}{}^{2}}$$

$$F_{z}=G_{1}/G_{0}$$

$$\Lambda \left(y\right)=\int_{0}^{\infty }y^{\bar{b}-1}e^{-y}dy$$

Therefore, the molecular weight distribution of the produced styrene polymers can be calculated from the leading moments of polymers by Equation (39). The number-average molecular weight $M_{n}$ and weight-average molecular weight $M_{w}$ can be easily obtained from the molecular weight distribution, and the polydispersity of the polymers can be calculated as $HI=M_{w}/M_{n}$.

Once the molecular weight distribution of the produced styrene polymers is obtained, it can be approximated by a B-spline model. In this simulation, ten third-order basis functions are applied to construct the B-spline model, and the basis functions are formulated as

(40) $$B(i,j)=\left\{ \begin{array}{ll} -2/w{\left(i\right)}^{2}\cdot {\left(j-y(i)-w(i)/2\right)}^{2}+0.5, & y(i)\leq y\leq y(i)+w(i)\\ 0, & otherwise \end{array}\right.$$

where i stands for the ith basis function of the B-spline model, j denotes the chain length of the dead polymers and w(⋅) is the width of the basis function, and

y(i)=1+200(i−1),i=1,2,…,10

$$w(i)=\left\{ \begin{array}{ll} 220, & i=1,2,\ldots ,9\\ 199, & i=10 \end{array}\right.$$

**Figure 3:**
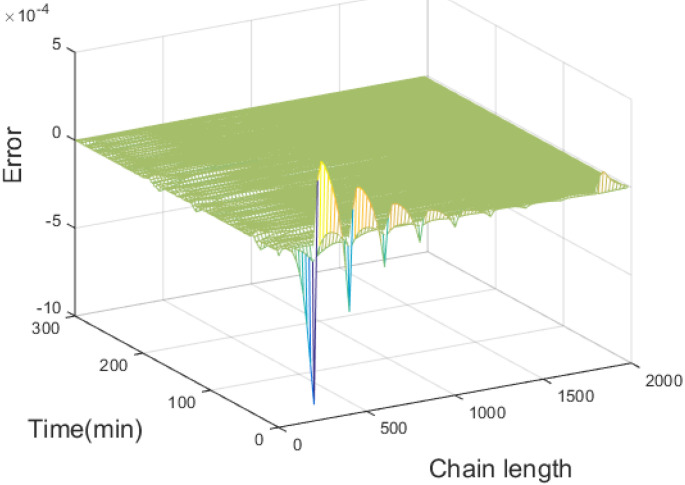
Approximation error of the B-spline model.

**Figure 4:**
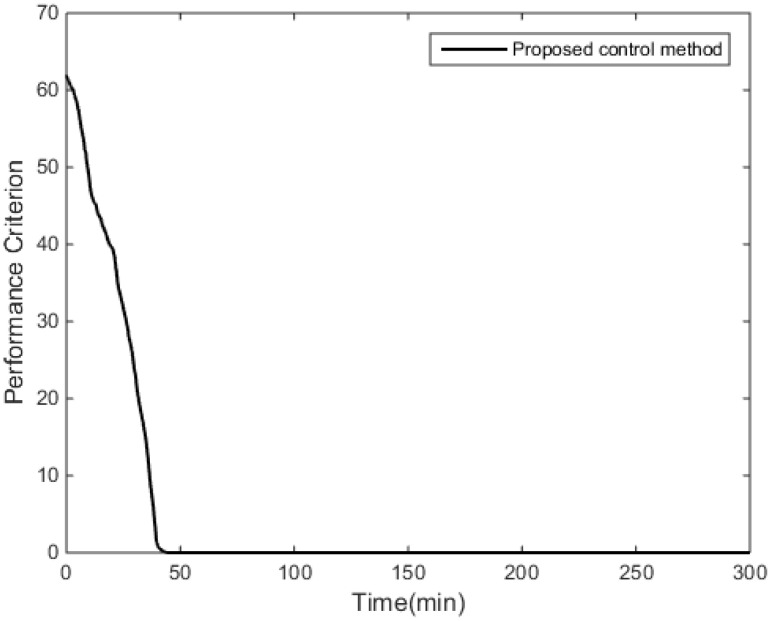
Value of the performance criterion.

The error of the B-spline approximation of the produced styrene polymer MWD is presented in Figure 3. It is obvious that the approximation error keeps within a proper range, indicating that the B-spline model is able to approximate the produced styrene polymer MWD effectively. The initial MWD, as shown in Figure 4, reaches the maximum value around chain length 200 and then gradually decreases; thus, the error in the model decreases with an increasing chain length.

### 4.2. MWD Shape Control of the Styrene Polymerization Process

**Figure 5:**
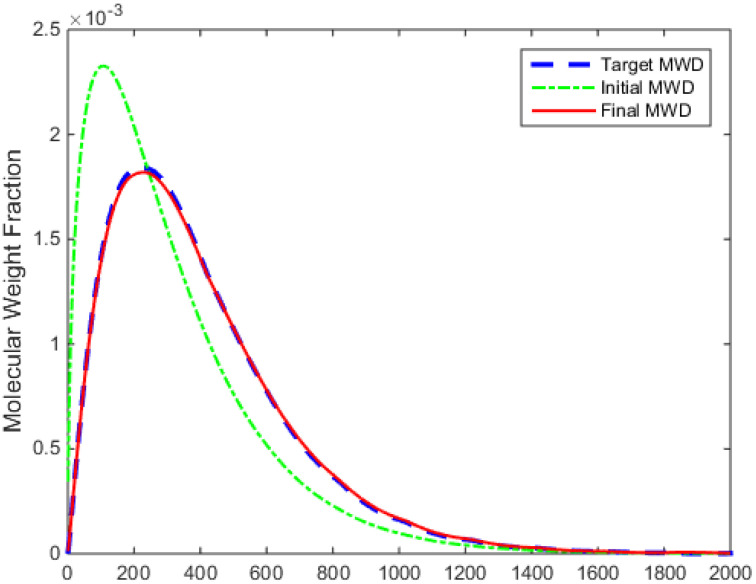
MWDs of styrene polymers at instants.

The control objective in this simulation is regulating the molecular weight distribution of the produced styrene polymers towards the target distribution, which is shown in Figure 5. The ratio, c, is the control input of the MWD shape control system for the styrene polymerization process. 

The sampling period is set to 1s, the initial input is 0.47 and for the weights of the performance criterion (27), Q is a 10-order identity matrix, and R=0.1.

**Figure 6:**
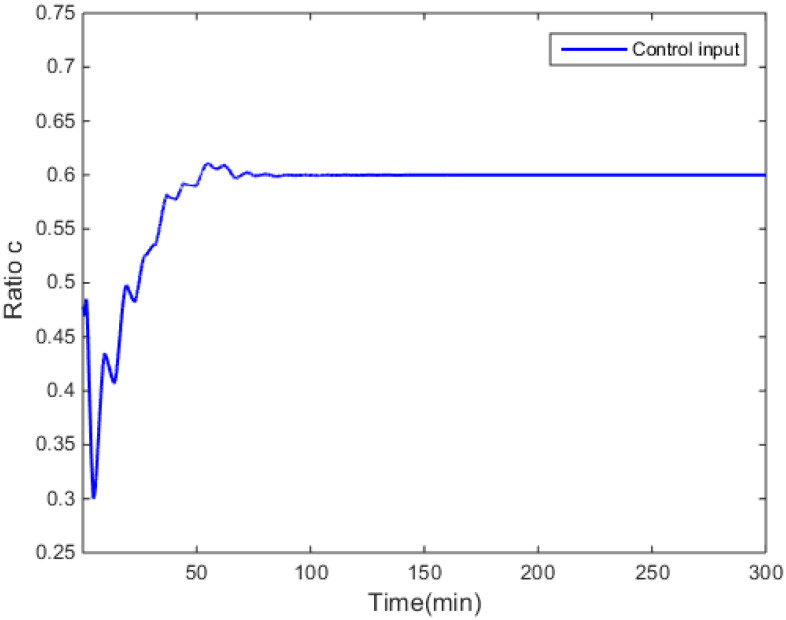
Ratio of the monomer flow rate over time.

**Figure 7:**
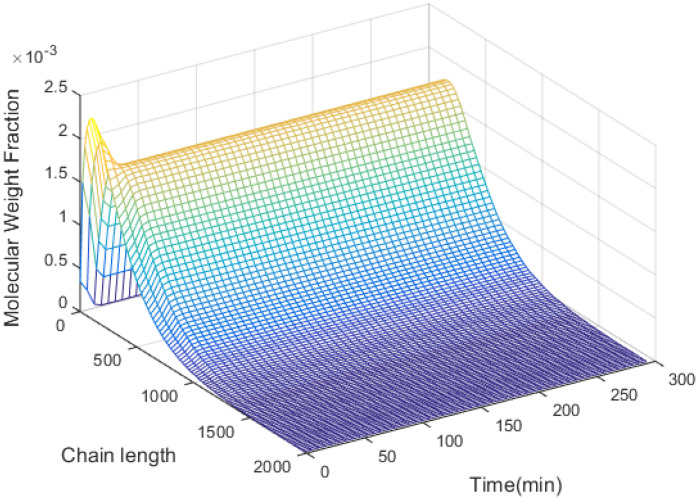
Three-dimensional MWD of styrene polymers.

**Figure 8:**
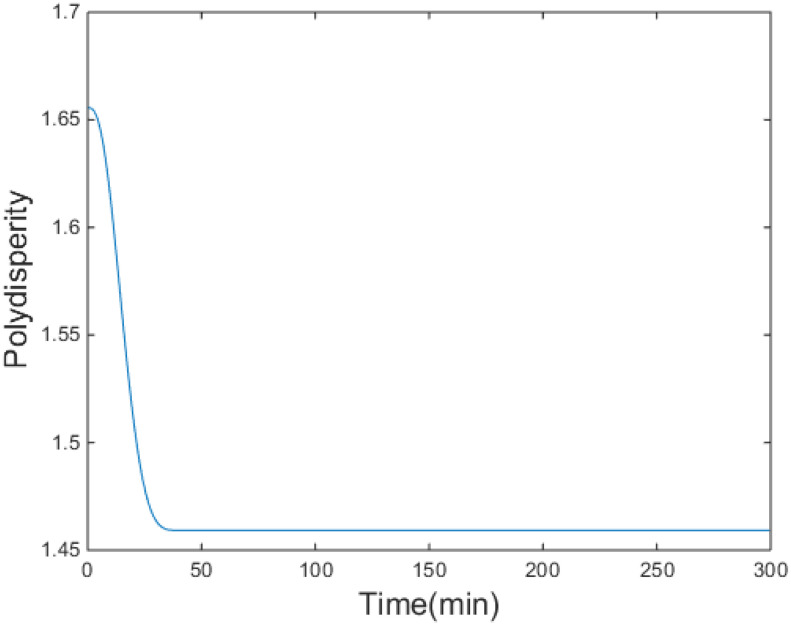
Polydispersity over time.

The simulation results are shown in Figure 4, Figure 5, Figure 6, Figure 7 and Figure 8.

Figure 4 demonstrates that the performance criterion can be regulated to a small value in a relatively short time by the proposed control method. The MWDs of the polymerization process at certain times are demonstrated in Figure 5. The variations of the ratio (manipulated variable of the control system) are illustrated in Figure 6, and the variations are within a proper range; the ratio keeps relatively stable when the MWD of the polymerization process is regulated close to the target one. The evolution of the MWD is illustrated by a three-dimensional graph in Figure 7. The change in the polydispersity over time is shown in Figure 8, which can be kept within a proper range. It can be seen that the MWD of the polymerization process gradually approaches the target MWD when the performance criterion gradually becomes smaller under the proposed algorithm.

The simulation results shown above illustrate that the moment-generating function-based control algorithm for polymerization processes’ MWD control is effective, and both the regulating time and the evolution of the MWD are satisfactory.

## 5. Conclusions

In this work, a control algorithm using the moment-generating function was presented for MWD shaping of polymerization processes. The output MWD was approximated by a discrete-time B-spline MWD model. Based on the input and expansion weight data of the B-spline model (which was used to approximate the output MWD), a system model could be identified through the N4SID method. Then, a performance criterion based on the moment-generating function was proposed, and the control algorithm could be derived by minimizing the performance criterion. The proposed control algorithm was tested in a styrene polymerization process, and the simulation results confirmed its effectiveness. The manipulated variable can be kept within a proper range, and the regulation time is satisfactory. Output MWD can also be adjusted to the target MWD gradually. The contributions can be summarized as:

1. The moment-generating function was used to construct the performance criterion, which simplifies the regulation of criterion weights so that the application of the proposed control method to the practical polymerization process becomes more convenient;
2. The subspace identification method was applied to establish the dynamic weights model of the controlled system based on the pair data of the control input and expansion weights, which avoids the numerical ill-conditioning and parameter overlap problems in traditional identification methods;
3. The proposed moment-generating, function-based control algorithm is effective for the MWD shaping of the styrene polymerization process. Both the evolution of the output MWD and the regulation time are satisfactory.

## Data Availability

Not applicable.
